# The Use of a Heterogeneously Controlled Mouse Population Reveals a Significant Correlation of Acute Phase Parasitemia with Mortality in Chagas Disease

**DOI:** 10.1371/journal.pone.0091640

**Published:** 2014-03-20

**Authors:** Tiago L. M. Sanches, Larissa D. Cunha, Grace K. Silva, Paulo M. M. Guedes, João Santana Silva, Dario S. Zamboni

**Affiliations:** 1 Department of Cell Biology, University of São Paulo, Medical School Ribeirão Preto, FMRP/USP, São Paulo, Brazil; 2 Department of Biochemistry and Immunology, University of São Paulo, Medical School Ribeirão Preto, FMRP/USP, São Paulo, Brazil; Instituto de Ciências Biomédicas/Universidade de São Paulo – USP, Brazil

## Abstract

Chagas disease develops upon infection with the protozoan parasite *Trypanosoma cruzi* and undergoes an acute phase characterized by massive parasite replication and the presence of parasites in the blood. This condition is known as acute phase parasitemia. This initial stage may result in a cure, in the development of the chronic stages of the disease or in the death of the infected host. Despite intensive investigation related to the characterization of the acute and chronic phases of the disease, the cause-effect relationship of acute phase parasitemia to the outcome of the disease is still poorly understood. In this study, we artificially generated a heterogeneously controlled mouse population by intercrossing F1 mice obtained from a parental breeding of highly susceptible A/J with highly resistant C57BL/6 mouse strains. This F2 population was infected and used to assess the correlation of acute phase parasitemia with the longevity of the animals. We used nonparametric statistical analyses and found a significant association between parasitemia and mortality. If males and females were evaluated separately, we found that the former were more susceptible to death, although parasitemia was similar in males and females. In females, we found a strong negative correlation between parasitemia and longevity. In males, however, additional factors independent of parasitemia may favor mouse mortality during the development of the disease. The correlations of acute phase parasitemia with mortality reported in this study may facilitate an appropriate prognostic approach to the disease in humans. Moreover, these results illustrate the complexity of the mammalian genetic traits that regulate host resistance during Chagas disease.

## Introduction

Chagas disease is a parasitic infection caused by the protozoan parasite *Trypanosoma cruzi* that causes strong morbidity effects in immunocompetent individuals. Despite its great impact on public health, this neglected tropical disease is still understudied, possibly because it primarily affects underprivileged populations [1]. The disease was originally endemic in Latin America and was confined to the Western Hemisphere. However, it has been spreading steadily to other continents and currently affects 8–10 million people worldwide, causing more than 10,000 deaths yearly [2]–[4]. Although human infection occurs primarily through vector-borne transmission, it may also occur via blood transfusions, organ donations, ingestion or transplacentally [4]–[6].

Two distinct phases characterize the course of disease development. A massive intracellular replication of *T. cruzi* occurs during an initial acute phase, characterized by the presence of trypomastigote forms of the parasite in the blood (known as acute phase parasitemia, herein termed parasitemia) and nonspecific clinical symptoms such as fever, occasional swelling and pain at the site of infection [7], [8]. Variation in the number of circulating parasites, including high peaks of parasitemia, can be observed during the acute phase. This initial stage of the disease can evolve to immunity-mediated cure of the infected patients, to death or to the development of the chronic stages of the disease [7]. Morbidity is dependent on the parasite strains and inoculation dose, but it is also associated with an inadequate protective immune response in certain risk groups of patients, including children, elderly and immunocompromised individuals [9]–[11]. In approximately one-third of the infected individuals, the disease evolves to a chronic phase triggered by parasite migration to muscle tissues, particularly the heart and the digestive tract. In most cases, the chronic phase of infection persists in latent and asymptomatic form for years or decades before the emergence of clinical symptoms such as arrhythmia, cardiomyopathy, heart failure, megaesophagus and megacolon [12].

The factors influencing the extension of the acute phase, death or transition to the chronic manifestation of Chagas disease are still poorly understood. Chagas disease is a complex disorder in which the parasite strain and the immunity of the host affect the development and manifestation of the disease. Therefore, it is well accepted that the genetic characteristics of both the parasite and the host are important in determining the outcome of infection [11], [13]–[16]. In this scenario, the reduction of the complexity of these interactions is essential to facilitate the experimental assessment of the factors influencing the course of Chagas disease. In this sense, different strains of *T. cruzi*, as well as traditionally established murine experimental models of infection, have effectively contributed to the examination of specific aspects of the pathogenesis of Chagas disease. Among the strains of *T. cruzi* that are widely used in scientific investigations are Y, CL, JG and Tulahuen, which have been characterized in terms of their infection in mouse models. These characteristics include, e.g., virulence, parasitemia and tissue tropism [17]–[22]. Among these strains, the Y strain of *T. cruzi* is highly virulent; it induces markedly high peaks of parasitemia in the acute phase of infection and causes fulminant death in certain inbred strains of mouse, e.g., A/J, C3H/HePas and C3H/HeN. In contrast, infection of the C57BL/6 mouse strain with the Y strain of *T. cruzi* results in an acute infection phase showing moderate-to-low levels of parasitemia and usually does not cause the death of the infected mice [22], [23]. Accordingly, the exploration of the features of the infection with the Y strain of the parasite in mice that are genetically deficient for certain genes in the C57BL/6 genetic background has revealed the importance of diverse receptors, chemokines and cytokines for resistance and susceptibility to Chagas disease [21], [24]–[29]. Nevertheless, the strategy of using isogenic parasites to infect isogenic mouse strains has produced no genetic variation and, therefore, has not allowed the evaluation of questions that are fundamental for the comprehensive understanding of Chagas disease. Among these pivotal unsolved questions are the relationship between parasitemia and the risk of death during the acute phase of infection, a feature that is not yet clear in the context of Chagas disease.

In this study, we addressed the correlation between parasitemia induced during the acute phase of infection and mortality during the course of infection in a mouse model of Chagas disease. To address this question, we artificially generated a F2 mouse population by intercrossing F1 mice obtained from a parental breeding of the highly susceptible A/J strain with the highly resistant C57BL/6 strain. This F2 population was infected with the highly virulent Y strain of *T. cruzi* and evaluated for mortality and for specific parameters related to parasitemia. Using nonparametric statistical analyses, we found a significant association between parasitemia and mortality. By analyzing males and females separately, we found that males were more susceptible to death but that parasitemia was similar in males and females. Accordingly, we found a strong negative correlation of parasitemia with longevity in females but not in males, suggesting that additional factors independent of parasitemia cause early mortality in males during infection with *T. cruzi*. The results of this study serve to elucidate a controversial issue underlying this disease: the statistical correlation of acute phase parasitemia with mortality during the outcome of Chagas disease.

## Materials and Methods

### Mouse strains and crossings

C57BL/6, A/J, F1 and F2 populations (generated in this study) were bred in the Animal Facilities of the Medical School Ribeirão Preto (FMRP-USP). Sixty-seven (C57BL/6× A/J) F2 progeny (n = 35 males and n = 32 females) were generated by systematic mating of siblings from (C57BL/6× A/J) F1. All animals were kept at 25°C with food and water *ad libitum.*

### Ethics Statement

The care of the mice was in compliance with the institutional guidelines on ethics in animal experiments; approved by CETEA (Comissão de Ética em Experimentação Animal da Faculdade de Medicina de Ribeirão Preto, approved protocol number 097/2010). CETEA follow the Brazilian national guidelines recommended by CONCEA (Conselho Nacional de Controle em Experimentação Animal).

### Parasites and infection

Eight- to ten-week-old mice were used for the infection experiments. The mice were infected i.p. with 10^3^ blood-derived trypomastigotes from the Y strain of *T. cruzi*. Survival rates were determined by inspection of the cages (twice a day) for 30 days after infection. Parasitemia was monitored by microscopic analysis of 5 µl blood samples drawn from the tail vein on 7, 9, 11 and 13 days after infection. The average, SD and peak values were used to describe the parasitemia counts during the analysis according to the following equations:
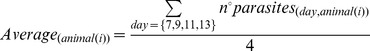


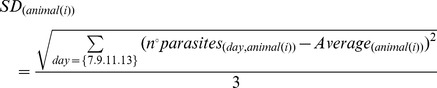


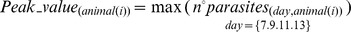



### Statistical analyses

The significance level was fixed at 0.05. Tests of normality were applied, namely, a Kolmogorov-Smirnov test and graphical analysis. Because the normality of the data was not confirmed, nonparametric statistical analyses were applied. A Mann-Whitney two-tailed test was applied to analyze possible differences between two groups; a Kruskal-Wallis one-way test was applied to three or more groups, with a Dunn multiple comparison test. A survival analysis was performed with the Kaplan-Meier estimator. The association of gender with the survival of infected mice was evaluated with a Mantel-Cox test. A test of the correlation between the mortality and parasitemia estimates was made with a Spearman correlation coefficient and a test for non-zero slope. Statistical significance was considered when *P*<0.007 for male individuals, when *P*<0.029 for females individuals, and when *P*<1.646×10^−5^ for total population, as estimated using a Spearman coefficient value of |0.5|. The statistical analyses were performed with GraphPad Prism 5.0 software.

## Results

### Generation of an artificially heterogeneous mouse population for parasitemia and mortality in response to *T. cruzi* infection

**Figure 1:**
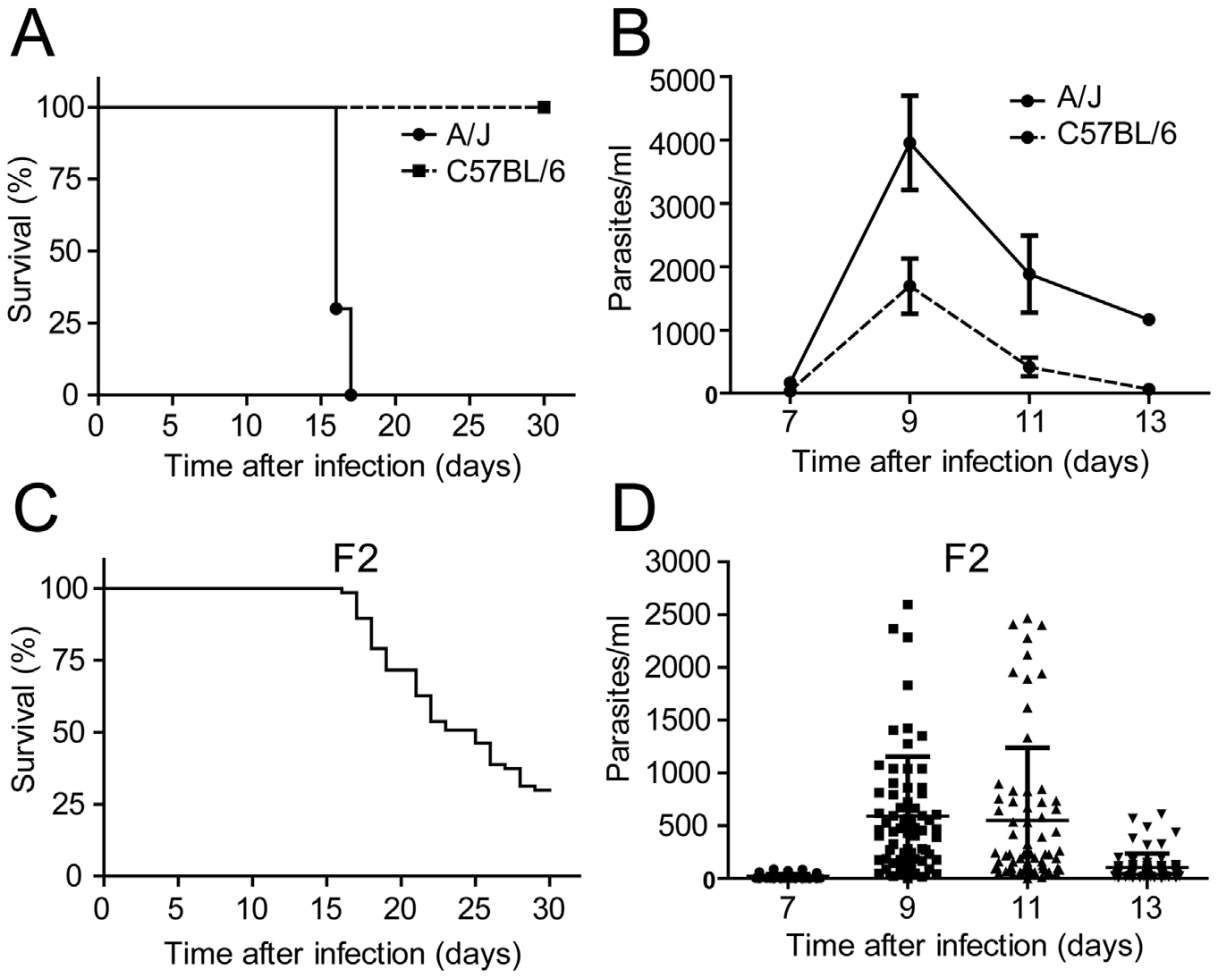
An artificially generated F2 offspring of an F1(C57BL/6× A/J) intercross is heterogeneous for mortality and parasitemia in response to infection with the Y strain of *T. cruzi*. Mice of parental resistant C57BL/6 and susceptible A/J strains and a F2 population composed of 67 individuals were infected i.p. with 1000 trypomastigotes. (A) Mortality of C57BL/6 (n = 10) and A/J (n = 10) mice was evaluated by daily inspection of cages. (B) Parasitemia of C57BL/6 and A/J mice was quantified microscopically by counting the parasites per ml of blood obtained from the tail vein at 7, 9, 11 and 13 days of infection. (C) Mortality of 67 F2 mice was evaluated by daily inspection of cages. (D) Parasitemia of 67 F2 mice was quantified microscopically by counting the parasites per ml of blood obtained from the tail vein at 7, 9, 11 and 13 days of infection. Each symbol in “D” represents an individual mouse, lines in “D” represent mean and bars in “B and D” represent standard deviation of the mean.

A correlation between parasitemia and mortality in the acute phase of Chagas disease is still controversial in the field. It is possible that definitive results have not previously been achieved because the resolution of this question requires a heterogeneous population of hosts to support appropriate analyses of the correlation between these parameters. To address the correlation between mortality and parasitemia in the outcome of *T. cruzi* infection, we generated an informative F2 population. This specific F2 population was generated by intercrossing littermates derived from the breeding of the highly susceptible A/J with the highly resistant C57BL/6 mouse strains. Divergence in susceptibility to *T. cruzi* infection has been previously reported for different inbred mouse strains [19], [23], [30]–[33]. In studies specifically addressing the susceptibility to the Y strain of *T. cruzi*, it has been shown that mice from the A/J inbred strain succumb to infection, whereas C57BL/6 mice survive to the acute phase when infected with low inoculum (∼1000 forms) [22], [23]. Under the conditions used in this study, we found that A/J mice infected with *T. cruzi* succumbed to infection during the acute phase, whereas all C57BL/6 infected mice survived during the same observation period (Fig. 1A). The contrasting differences in the survival of infection between these two mouse strains were similarly observed for the parasitemia of infected mice. A/J infected mice showed a higher peak of parasitemia in the course of the acute phase of infection relative to C57BL/6 mice (Fig. 1B). The survival and parasitemia of an F2 population derived from a parental intercross between A/J and C57BL/6 mice were evaluated. We found a variable rate of mortality in the infected F2 population: of the 67 infected F2 mice, 47 succumbed to infection, whereas the remaining mice (considered survivors) survived until day 30 after infection (Fig. 1C). The analysis of individual parasitemia in the 67 infected F2 mice showed that a pronounced heterogeneity in parasite counts occurred on the days of higher parasite counts (i.e., 9 and 11 days after infection), with massive variations in the parasite levels in the blood of the infected mice (Fig. 1D). These results indicate that the F2 population generated in this study showed marked variability in survival and parasitemia in response to infection with the Y strain of *T. cruzi*. This heterogeneous mouse population provided a level of individual variation necessary for the further assessment of the correlation between survival and parasitemia in a controlled cohort of animals with experimentally induced Chagas disease.

### Evaluation of parameters to describe parasitemia in mice infected with *T. cruzi*

**Figure 2:**
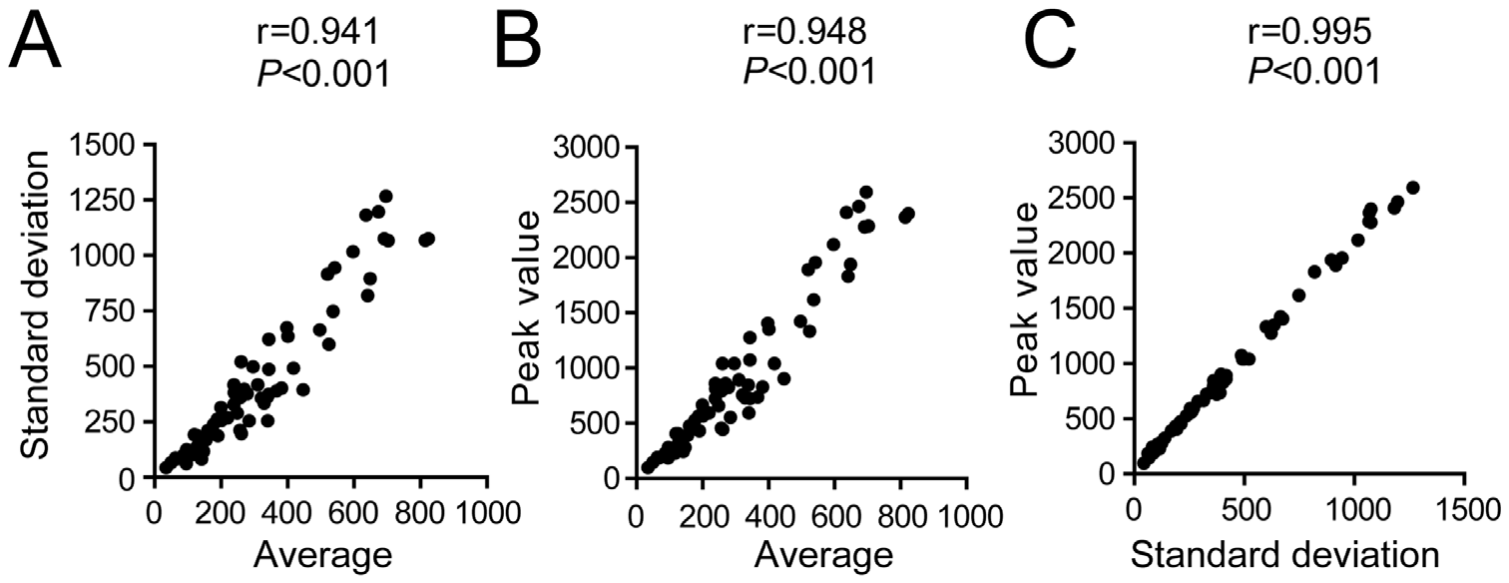
Distinct quantitative parameters can be used to assess parasitemia of infected F2 mice. Parasitemia values (7, 9, 11 and 13 days after infection) of the 67 F2 mice infected with the Y strain of *T. cruzi* (same mice shown in Fig. 1D) were used to generate distinct parameters to assess parasitemia. (A) Correlation of the average number of parasites per ml of blood at days 7, 9, 11 and 13 of infection (Average) with the standard deviation of the parasitemia mean at days 7, 9, 11 and 13 of infection (Standard deviation). (B) Correlation of the Average with the value of parasitemia obtained at the day of the parasitemia peak (Peak value). (C) Correlation of the Standard deviation with the Peak value. Correlation was assessed with a Spearman test. A test of non-zero slope was applied, and the *P* value and r are shown. Correlation was considered significant if *P*<0.05.

Initially, we sought to explore various parameters associated with the course of parasitemia to identify those parameters that appropriately described individual parasitemia during the acute phase of infection. As shown in Fig. 1D, the parasitemia of the 67 F2 mice infected with *T. cruzi* was systematically measured on days 7, 9, 11 and 13 after infection. We evaluated specific estimators that aggregated the information on parasitemia from days 7, 9, 11 and 13 in a single value that depicted the parasitemia of each individual mouse. The first parameter used was the average of parasitemia on days 7, 9, 11 and 13 (termed “Average”). This parameter reflects the average number of circulating parasites per mouse during the acute phase of infection. The second parameter was the square root of the variance of parasitemia at days 7, 9, 11 and 13 (termed “Standard deviation”). This parameter reflects the variability in parasitemia among the 4 days of measurement for each mouse. The third parameter was the value of parasitemia detected on the day of the parasite peak (termed “Peak value”). This parameter reflects the highest value of parasitemia found in each mouse, regardless of the day after infection. To further determine whether all the chosen parameters effectively reported the parasitemia for each individual mouse, we calculated the correlations between the parameters. We found that all of the selected parameters were strongly correlated with each other (Fig. 2). We noted that the Average for each mouse was strongly correlated with the Standard deviation (r = 0.941, Fig. 2A). The strongest correlation detected was that between the Peak value and the Standard deviation (r = 0.995, Fig. 2C). Note that the significant correlations obtained among the three parameters indicate that these parameters are appropriate to express the parasitemia of each individual mouse. For this reason, we proceeded with the analyses using the Average, Standard deviation, and Peak value as the parameters to describe parasitemia.

### Association between parasitemia and mortality in F2 mice infected with *T. cruzi*

**Figure 3:**
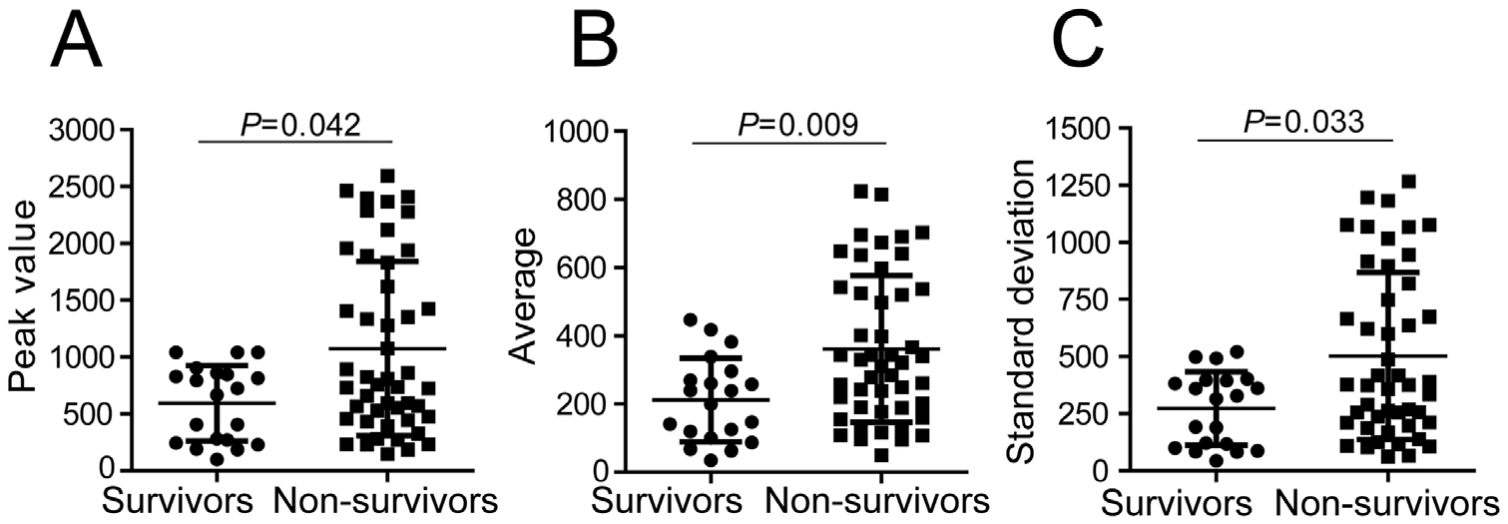
Mortality of F2 mice infected with *T. cruzi* is associated with parasitemia. Parasitemia of the 67 F2 mice infected with the Y strain of *T. cruzi* (same mice shown in Fig. 1D) was indicated for survivors (n = 20 F2 mice) and non-survivors (n = 47 F2 mice). The value of parasitemia obtained at the day of the parasitemia peak (Peak value; A), the average number of parasites per ml of blood at days 7, 9, 11 and 13 of infection (Average; B) and the standard deviation of the parasitemia mean at days 7, 9, 11 and 13 of infection (Standard deviation; C) was indicated for survivors and non-survivor F2 mice. Each symbol represents an individual mouse, lines and bars represent mean and standard deviation of the mean. Association was considered significant if *P*<0.05 (Mann-Whitney test).

Initially, we divided all 67 F2 mice into those that survived (n = 20, termed “survivors”) and those that succumbed to *T. cruzi* infection (n = 47, termed “non-survivors”). According to this categorization, we found that the non-survival group was associated with a higher level of parasitemia, as demonstrated by the higher Peak value, Average and Standard deviation (Fig. 3A–C, respectively). Note that we found individuals with remarkably low parasitemia within the group of non-survivors, indicating that, regardless of the significant association of parasitemia with mortality, the development of a reduced parasitemia does not guarantee survival.

**Figure 4:**
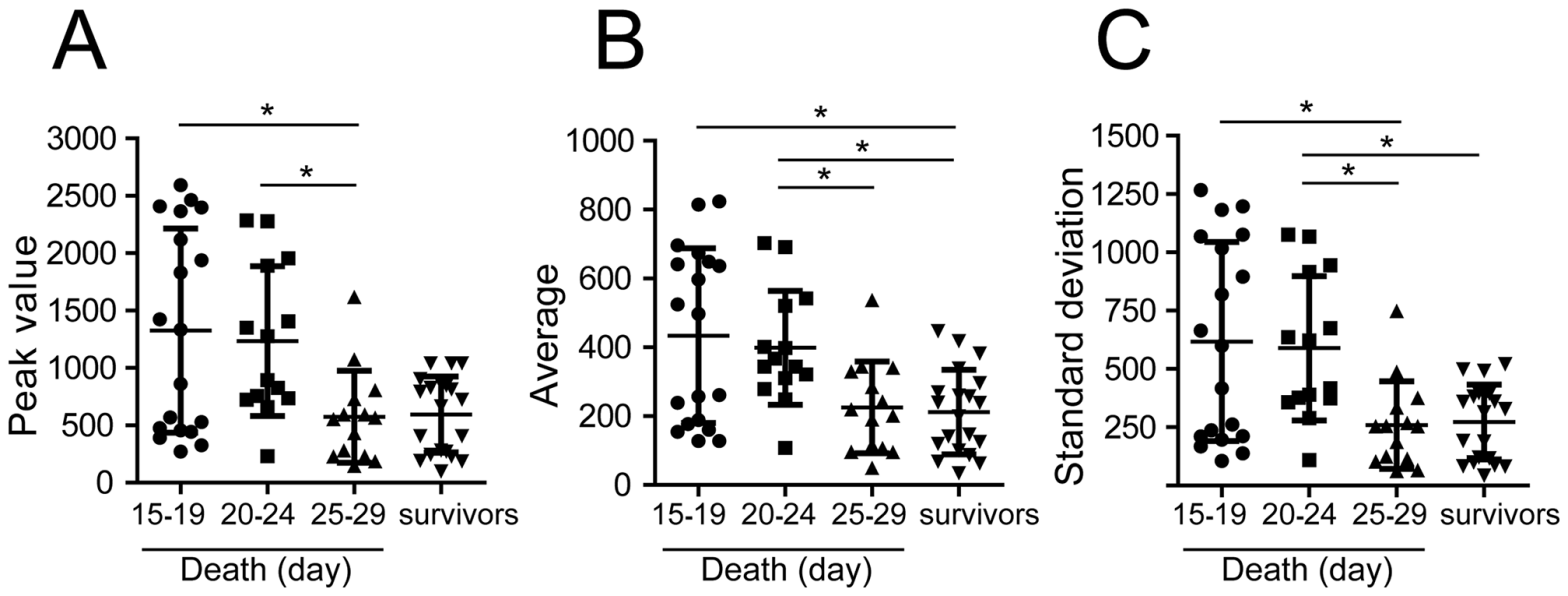
The longevity of non-survivors is associated with parasitemia. Parasitemia of the 67 F2 mice infected with the Y strain of *T. cruzi* (same mice shown in Fig. 1D) was categorized according to the day of death: 15–19 (n = 19 mice), 20–24 (n = 14 mice) and 25–29 (n = 14 mice) days after infection. The survivors are also indicated (n = 20). The value of parasitemia obtained at the day of the parasitemia peak (Peak value; A), the average number of parasites per ml of blood at days 7, 9, 11 and 13 of infection (Average; B), and the standard deviation of the parasitemia mean at days 7, 9, 11 and 13 of infection (Standard deviation; C) was indicated for the survivors and categorized non-survivors in the F2 mice. Each symbol represents an individual mouse, lines and bars represent mean and standard deviation of the mean. Association was considered significant if *P*<0.05 based on a Kruskal-Wallis test and a Dunn post-test.

Next, we categorized the group of non-survivors by the day of death. Therefore, the non-survivor group was subdivided into three groups: those that died early (between days 15–19, n = 19 mice), those that died at an intermediate time (between days 20–24, n = 14 mice) and those that died late (between days 25–29, n = 14 mice) after infection. We noticed that the mice with strikingly higher parasitemia succumbed to infection significantly earlier (died between days 15–19 or 20–24 after infection) in comparison with the group with lower parasitemia (died between 25–29 days) (Fig. 4A–C). Note that the parasitemia of the group that died late (between days 25–29) did not differ from that of the group of survivors. Collectively, these data support the hypothesis that mortality is indeed associated with parasitemia and that the longevity of the deceased mice was inversely correlated with parasitemia.

A careful examination of the distribution of parasitemia in the group that died between days 15–19 indicates that the parasitemia measured in this group of mice followed a bimodal distribution. That is, certain animals presented a remarkably low level of parasitemia, whereas other animals presented an extremely high level of parasitemia. To further evaluate the possible influence of the sex of the animals on this particular distribution, we analyzed the parasitemia in this group, separating the males from the females. Interestingly, the group with high parasitemia (more than 1000 parasites in the Fig. 4A) included both males and females (5 males and 5 females), whereas the mice showing low parasitemia (less than 1000 parasites in the Fig. 4A) were composed by 8 males and one female (data not shown). This observation induced us to further investigate the putative role of gender in the correlation between parasitemia and mortality.

### Sex influences survival but not parasitemia in F2 mice infected with *T. cruzi*

**Figure 5:**
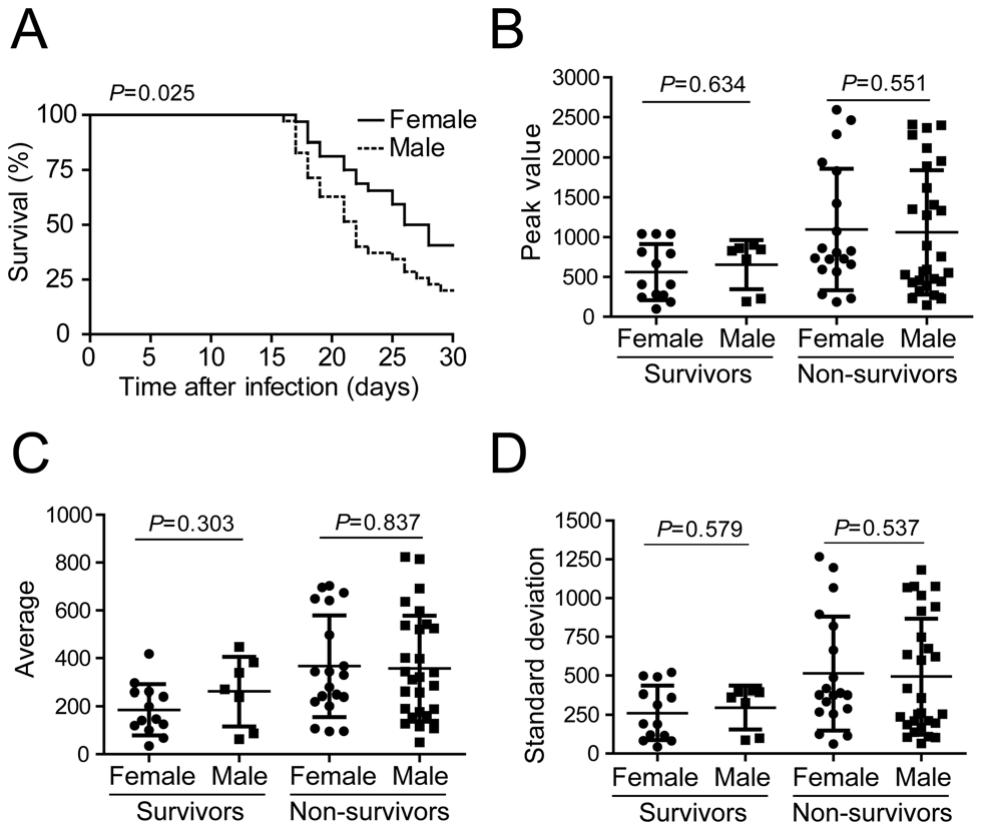
Survival but not parasitemia of F2 mice is a trait influenced by sex. (A) Survival curve of females (n = 32) and males (n = 35) of the 67 F2 mice infected with the Y strain of *T. cruzi* (same mice shown in Fig. 1D). (B–D) Parasitemia of the 67 F2 mice infected with the Y strain of *T. cruzi* (same mice shown in Fig. 1D) was associated with the survival status of individuals categorized by gender. The value of parasitemia obtained at the day of the parasitemia peak (Peak value; B), the average number of parasites per ml of blood at days 7, 9, 11 and 13 of infection (Average; C) and the standard deviation of the parasitemia mean at days 7, 9, 11 and 13 of infection (Standard deviation; D) was indicated by gender for survivors and non-survivor F2 mice. Each symbol represents an individual mouse, lines and bars represent mean and standard deviation of the mean. Association was considered significant if *P*<0.05. Mantel-Cox test (A) and Mann-Whitney test (B–D).

To further investigate the influence of sex on the correlations obtained in this study, we first analyzed possible differences in survival rate between the males and females in the F2 population. As shown in Fig. 5A, the F2 females were more resistant to infection overall than the males (*P* = 0.025, n = 35 males and n = 32 females). By comparing the parasitemia of males vs. females for both the survivors and the non-survivors, we found no significant differences in parasitemia between the survivors and the non-survivors (Fig. 5B–D). Collectively, these data indicate that the sex of the individual mouse may impact mortality but does not influence the development of parasitemia during infection with the Y strain of *T. cruzi*.

### The longevity of mice after *T. cruzi* infection is inversely correlated with parasitemia in females but not in males

**Figure 6:**
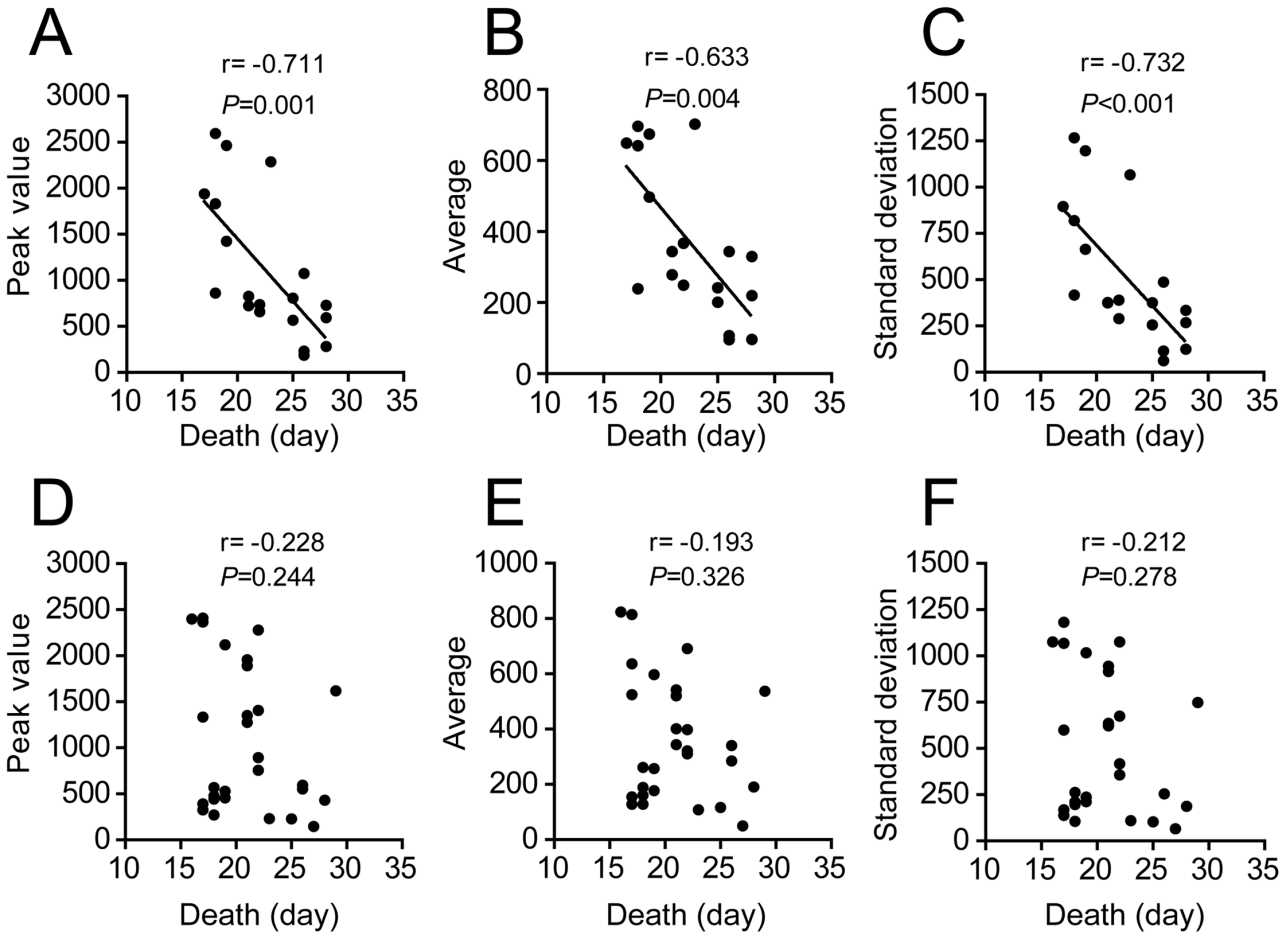
Parasitemia is inversely correlated with longevity in female but not in male mice of the F2 population. Parasitemia of the 47 F2 mice that succumbed to infection with the Y strain of *T. cruzi* (same shown in Fig. 1D) was correlated with longevity according to the sex of the mice. The value of parasitemia obtained at the day of the parasitemia peak (Peak value; A, D), the average number of parasites per ml of blood at days 7, 9, 11 and 13 of infection (Average; B, E), and the standard deviation of the parasitemia mean at days 7, 9, 11 and 13 of infection (Standard deviation; C, F) was correlated with the day of death of the non-survivor F2 mice. The females (n = 19, A–C) and males (n = 28; D–F) are indicated. Correlation was assessed with a Spearman test. A test of non-zero slope was applied, and the *P* value and r are shown. Correlation was considered significant if *P*<0.05.

The data reported above indicate that survival rates differed between males and females of the F2 population (Fig. 5A). Moreover, we identified a male population showing short longevity and low parasitemia (Fig. 4D–E). Therefore, we hypothesized that the analysis of the correlation between mortality and parasitemia would differ if we analyzed only animals of the same sex. We found that the correlation between mortality and parasitemia was statistically significant and highly robust if we analyzed only the female population (Fig. 6A–C). In contrast, we found no significant correlation if we analyzed only the male population (Fig. 6D–E). Collectively, these results demonstrate that parasitemia was inversely correlated with longevity. However, this correlation was only significant for females. It is possible that specific factors present in the genotype of the male mice caused an early death in these animals, independent of the parasitemia.

## Discussion

Chagas disease is an important human health problem in the southern regions of the Western Hemisphere and is progressively spreading worldwide. Understanding the genetic traits of the host and parasite that play a role in the outcome of infection with *T. cruzi* is pivotal to an appropriate prognostic approach to the disease and for the development of effective treatments. Although the development of acute phase parasitemia in experimental models of Chagas disease has generally been overlooked as a parameter associated with susceptibility to infection, the importance of acute phase parasitemia for the development, severity and outcome of Chagas disease is still unclear. In this study, we developed a controlled, yet genetically heterogeneous mouse population to address the correlation pattern of parasitemia and longevity in an experimental model of Chagas disease. By systematically infecting this heterogeneous population with the Y strain of *T. cruzi*, we demonstrated a significant association between parasitemia and mortality. However, acute phase parasitemia is not the sole feature that causes the death of infected mice, as we identified a population that developed a remarkably low level of parasitemia and succumbed to infection between days 15 and 19 after infection (Fig. 6). Therefore, our data support the hypothesis that the development of elevated parasitemia during the acute phase of the disease may favor the death of the infected host. Nevertheless, a reduced level of parasitemia during the acute phase of the disease does not guarantee survival. This principle is particularly evident in the population of male mice because the males constituted the group of mice that developed a remarkably low level of parasitemia and succumbed to infection soon after infection.

It is widely known that the sex of the individual can influence or determine the phenotypic expression of host genetic traits controlling the susceptibility of mice to infection. In the case of the response against parasites, sexual dimorphism involves, for example, the modulation of immune responses by sex steroids, thus directly influencing parasite control [34], [35]. Susceptibility *loci* on the murine X chromosome have been recently identified for *Plasmodium chabaudi*. A locus designated as Char11 was identified via segregation analysis of a F2 progeny generated from SM/J (susceptible) and C57BL/6J (resistant) parental strains [36]. In the case of *Leishmania major*, the locus lmr3 on the X chromosome has been shown to be epistatically controlled by the autosomal locus lmr1 on chromosome 9 and to influence susceptibility to infection in a genetic linkage mapping using reciprocally intercrossed F2 populations from BALB/c (susceptible) and C57BL/6 (resistant) strains [37]. For *T. congolense*, evidence suggests that genomic imprinting controls Tir1, a major locus involved in mouse susceptibility to the parasite [38]. Finally, in the case of *T. cruzi*, it has been shown that an X-linked mutation of Balb.*Xid* immunodeficient mice influences murine resistance to infection, this process may be dependent on the increased production of IFN-γ, which possibly account for the increased resistance of the BALB.XID as compared to Balb/c mouse [32], [39]. Accordingly, the effect of sex has also been reported to be important for susceptibility to Chagas disease, both in mouse and human infection [16], [22], [32], [39]–[41].

The effect of acute phase parasitemia on the outcome of the disease is still not clear in the field of Chagas disease. Using an experimental mouse model of Chagas disease, several authors have reported concomitant reductions in parasitemia and mortality in response to treatment with specific compounds or pharmacological agents [42]–[48]. Nevertheless, these studies fail to provide evidence that supports a cause-effect relationship between parasitemia and mortality. Indeed, other studies using a different inbred strain of mouse and a distinct parasite strain did not support a strong effect of acute phase parasitemia on the mortality of the infected animals [17], [49], [50]. Although these studies employed mice and/or parasites with heterogeneous variations, the number of distinct mouse and/or parasite strains was too low to support an appropriate statistical association of parasitemia and mortality. In this context, our heterogeneously controlled F2 population composed of 67 mice provided an appropriate experimental group that supported the assessment of the association of parasitemia with mortality in response to infection with *T. cruzi*. This population successfully supported analyses resulting in the conclusion that the development of acute phase parasitemia was indeed linked to the mortality and longevity of the infected mice. As expected for complex diseases such as Chagas, additional factors, regardless of parasitemia, also influenced the outcome of the disease. Importantly, the heterogeneity observed in this controlled F2 population, which is necessary for such studies, may be useful for further investigations aiming at the identification of specific genetic traits of the host that influence susceptibility to infection with *T. cruzi*.

Collectively, our data show a significant negative correlation of mouse longevity with parasitemia during the acute phase of *T. cruzi* infection. This correlation is robust if we analyze the females separately (Fig. 6). It is possible that the specific male population that presented remarkably low parasitemia and succumbed soon after infection (between days 15 and 19) interfered with the detection of statistically significant correlations between parasitemia and mortality in male mice (Fig. 6D–F). In contrast, analyses of the female population, which did not include a clearly defined population that developed low parasitemia and died soon after infection, resulted in a robust correlation between parasitemia and mortality (Fig. 6A–C). Of note, we have recently identified significant traits within the mouse X chromosome influencing survival [22]. In the A/J mouse background, the genes encoded in this chromosome may not be as functional as those encoded by C57BL/6 Chromosome X [22]. Therefore, it is possible that the male mouse population that displays low parasitemia and reduced longevity has inherited an A/J-derived X chromosome. Regardless of these yet-unidentified genes on chromosome X, our data clarify a long-lasting controversy in the field of Chagas disease: the association of acute phase parasitemia with longevity in individuals infected with *T. cruzi*. The identification of the correlation of parasitemia with early mortality in mammalian hosts contributes to our global understanding of the pathogenesis of this disease and provides additional relevant information to the prognostic to human Chagas disease.
